# Repertoires of Spike Avalanches Are Modulated by Behavior and Novelty

**DOI:** 10.3389/fncir.2016.00016

**Published:** 2016-03-22

**Authors:** Tiago L. Ribeiro, Sidarta Ribeiro, Mauro Copelli

**Affiliations:** ^1^Section on Critical Brain Dynamics, National Institute of Mental Health (NIMH), National Institutes of Health (NIH)Bethesda, MD, USA; ^2^Physics Department, Federal University of Pernambuco (UFPE)Recife, PE, Brazil; ^3^Brain Institute, Federal University of Rio Grande do Norte (UFRN)Natal, RN, Brazil

**Keywords:** avalanches, spikes, patterns, criticality, memory, novelty, sleep

## Abstract

Neuronal avalanches measured as consecutive bouts of thresholded field potentials represent a statistical signature that the brain operates near a critical point. In theory, criticality optimizes stimulus sensitivity, information transmission, computational capability and mnemonic repertoires size. Field potential avalanches recorded via multielectrode arrays from cortical slice cultures are repeatable spatiotemporal activity patterns. It remains unclear whether avalanches of action potentials observed in forebrain regions of freely-behaving rats also form recursive repertoires, and whether these have any behavioral relevance. Here, we show that spike avalanches, recorded from hippocampus (HP) and sensory neocortex of freely-behaving rats, constitute distinct families of recursive spatiotemporal patterns. A significant number of those patterns were specific to a behavioral state. Although avalanches produced during sleep were mostly similar to others that occurred during waking, the repertoire of patterns recruited during sleep differed significantly from that of waking. More importantly, exposure to novel objects increased the rate at which new patterns arose, also leading to changes in post-exposure repertoires, which were significantly different from those before the exposure. A significant number of families occurred exclusively during periods of whisker contact with objects, but few were associated with specific objects. Altogether, the results provide original evidence linking behavior and criticality at the spike level: spike avalanches form repertoires that emerge in waking, recur during sleep, are diversified by novelty and contribute to object representation.

## Introduction

Neuronal avalanches are spatiotemporal bouts of electrical activity that were first described at the level of local field potentials (LFP) as clusters of peak-valley oscillations with highly variable sizes and durations in cortical slices (Beggs and Plenz, 2003). Since its initial conception, neuronal avalanches have been demonstrated to permeate a striking number of highly different systems: from anesthetized rats (Gireesh and Plenz, 2008) and cats (Hahn et al., 2010), and awake monkeys (Petermann et al., 2009) using LFP or, more recently, in mice using voltage imaging (Scott et al., 2014) and two-photon imaging (Bellay et al., 2015), and in humans using electrocorticogram (Solovey et al., 2012), functional magnetic resonance imaging (Fraiman and Chialvo, 2012; Tagliazucchi et al., 2012; Haimovici et al., 2013), electroencephalogram (Meisel et al., 2013) and magnetoencephalogram (Palva et al., 2013; Shriki et al., 2013). The characteristic high variability of neuronal avalanches, reflected in power-law distributions of both sizes and durations, is held as evidence that they represent a link between brain activity and criticality. It has been shown that a critical brain would have desirable advantages such as optimal computational capabilities (Bertschinger and Natschläger, 2004), information transmission (Beggs and Plenz, 2003; Rämö et al., 2007) and diversity (Nykter et al., 2008), size of memory repertoires (Beggs and Plenz, 2004; Haldeman and Beggs, 2005) and sensitivity to stimuli (Kinouchi and Copelli, 2006; Shew et al., 2009; Gautam et al., 2015). For detailed reviews, see Chialvo (2010), Shew and Plenz (2013) and Hesse and Gross (2014).

These results raised great interest in this particular organization of neuronal activity. However, most of the work in the field still consists of detecting neuronal avalanches under resting conditions or from ongoing activity data, and only recently avalanches started to be studied under stimulus conditions (Shew et al., 2015). The question that naturally follows is whether these scale-free spatiotemporal activity cascades take part on information transmission and storage. That is to say, while neuronal avalanches represent a link between criticality and brain dynamics, their large variation in size and duration could imply a lack of reliability necessary for information storage. Yet, Scarpetta and de Candia (2013) have shown through computational analysis that neuronal avalanches are at the critical point between replay and non-replay of spatiotemporal activity patterns. Thus, if neuronal avalanches indeed constitute a fundamental unit of representation in the brain, they should conform to distinct and stable patterns of *in vivo* activity. In fact, Beggs and Plenz (2004) showed that LFP avalanches *in vitro* are diverse and precise activity patterns, repeatable for many hours in slice cultures. This finding strengthened the possibility that neuronal avalanches provide a spatiotemporal support for spike patterns that may subserve information encoding. Surprisingly, however, no further studies were performed to show that these results hold true for *in vivo* conditions.

Although recurring spike patterns have been subject of research for a long time (Abeles et al., 1993; Wilson and McNaughton, 1994; Nádasdy et al., 1999; Dave and Margoliash, 2000; Hoffman and McNaughton, 2002; Hahnloser et al., 2003; Luczak et al., 2007; Madhavan et al., 2007; Rolston et al., 2007; Pastalkova et al., 2008), similar investigations are absent for spike avalanches, characterized by uninterrupted activity over consecutive temporal bins (see “Materials and Methods” Section). Those avalanches were observed in dissociated neurons (Mazzoni et al., 2007; Pasquale et al., 2008), cell cultures (Tetzlaff et al., 2010; Vincent et al., 2012), forebrain regions of freely-behaving rats (Ribeiro et al., 2010), hippocampal cells of rats performing an open-field task, neurons of the primary visual cortex (V1) of anesthetized cats, and neurons from the prefrontal cortex of monkeys performing a visual short memory task (Priesemann et al., 2014). Statistical signatures of criticality were obtained in all those scenarios. While LFP avalanches represent the propagation of regionally synchronized activity, spike avalanches derive from individual spiking neurons. Therefore, regarding pattern repetition and information encoding, it is possible that these two types of avalanche propagation in the brain may play different roles. Finding repeating spike avalanches would also provide a missing link between those studies of recurring spiking activity and criticality, since work in this direction has been so far restricted to simulations (Haldeman and Beggs, 2005; Chen et al., 2010; Scarpetta and de Candia, 2013) or reduced preparations using LFP (Beggs and Plenz, 2004; Stewart and Plenz, 2006; Chen et al., 2010). Therefore, it remains to be determined whether spike avalanches form stable and repeatable repertoires in freely-behaving animals. Also unclear is the extent to which these putative repertoires reflect behavior and are modifiable by novel experience. To address these issues, we used the methods described by Beggs and Plenz (2004) to assess pattern repetition in 12 sets of ~40,000 spike avalanches recorded from the hippocampus (HP), primary somatosensory cortex (S1) or V1 of six freely-behaving rats spontaneously cycling between waking (WK), slow-wave sleep (SWS) and rapid-eye-movement sleep (REM). Recordings of action potentials (spikes) with chronically implanted multielectrode arrays were performed before (PRE), during (EXP) and after (POST) a ~20 min exposure to novel objects: ball, brush, urchin and food (Ribeiro et al., 2010) (see “Materials and Methods” Section). Avalanches were obtained separately for each brain region (HP, S1 or V1) across the experiment, and were classified according to the behavioral state (WK, SWS or REM) and stage of the experiment (PRE, EXP or POST). We refer to the set of avalanches obtained from one brain region of one animal as a sample. The statistics of the avalanches analyzed here were previously shown to be consistent to what is found in self-organized critical systems (Ribeiro et al., 2010). For example, after rescaling the waiting-time distributions for different avalanche sizes by the average waiting-time for each case, the distributions collapse together in a double power law function similar to what is found for earthquakes (Christensen et al., 2002). Moreover, the system displayed universality, with a single scaling function applying to the three different behavioral states, brain regions and stages of the experiment. Here, we focus on the repetitive nature of those avalanches, the relation to behavior, and the consequences for information encoding.

## Materials and Methods

### Multielectrode Recordings and Experimental Design

The experimental data analyzed here came from a previous study (Ribeiro et al., 2004). In brief, the animals were exposed to four novel objects (ball, brush, urchin and food), which were introduced at the corners of the recording box and left for about 20 min for free exploration. Recordings were performed before, during and after exposure. Visible lights were kept off throughout the experiment. Detailed information regarding neuronal recordings and LFP-based classification of major behavioral states can be found in the original study (Ribeiro et al., 2004).

All animal work including housing, surgical and recording procedures were conducted in strict accordance with the National Institutes of Health (NIH) guidelines, and the Duke University Institutional Animal Care and Use Committee, and was approved by the Edmond and Lily Safra International Institute of Neuroscience of Natal Committee for Ethics in Animal Experimentation (permit #04/2009).

### Spike Avalanches

In order to define a spike avalanche, the spike time series from all neurons were divided in bins of duration Δt (frames). The beginning of a neuronal avalanche is formally defined by the occurrence of a frame without any spikes (in any neuron) followed by a frame with at least one spike (in at least one neuron). The end of the avalanche is reached when another empty frame occurs (Beggs and Plenz, 2003). To rule out a systematic bias owing to the choice of time bin, we employed the same heuristic prescription as that of Beggs and Plenz (2003), namely to create a pooled time series with spikes from all neurons, and to use as time bin Δt the average inter-event interval, i.e., the time between consecutive spikes (whether or not from the same neuron). These rate-normalized time bins were therefore independently determined by the data, being specifically calculated for different samples, corresponding to a certain brain region (HP, primary somatosensory (S1) or visual (V1) cortices) of a given animal. Table 1 shows Δt values for each sample.

**Table 1:** **Avalanche and family data per sample**.

Sample (Animal/Brain region)	Temporal bin Δt (ms)	Minimum avalanche duration	Number of families	Number of significant families	Number of setlists (WK/SWS)	Maximum *p* value (significant families)	Estimate of false positives
GE1/HP	5.45	3	13771	1865	36/14	0.013	185
GE5/HP	5.94	3	16390	858	48/10	0.005	86
GE6/HP	3.26	5	12016	1322	42/8	0.011	132
GE17/HP	7.13	3	14321	375	50/11	0.0026	37
GE3/S1	3.89	4	14193	361	40/18	0.0025	35
GE4/S1	9.35	3	10051	0	Ø	Ø	Ø
GE5/S1	4.83	4	13282	246	48/10	0.0018	24
GE6/S1	1.98	6	17532	0	Ø	Ø	Ø
GE3/V1	2.86	5	16434	0	Ø	Ø	Ø
GE4/V1	4.03	5	12924	81	44/5	0.0006	8
GE5/V1	3.57	5	13374	208	48/10	0.0015	20
GE17/V1	2.28	6	10976	1874	50/11	0.017	186

*Note. The samples are ordered as in Figures 2, 3, 4, 5*.

We followed the methods introduced by Beggs and Plenz (2004) for avalanche pattern analysis. In the following sections we briefly describe the main steps of the procedure.

### Avalanche Comparison

**Figure 1:**
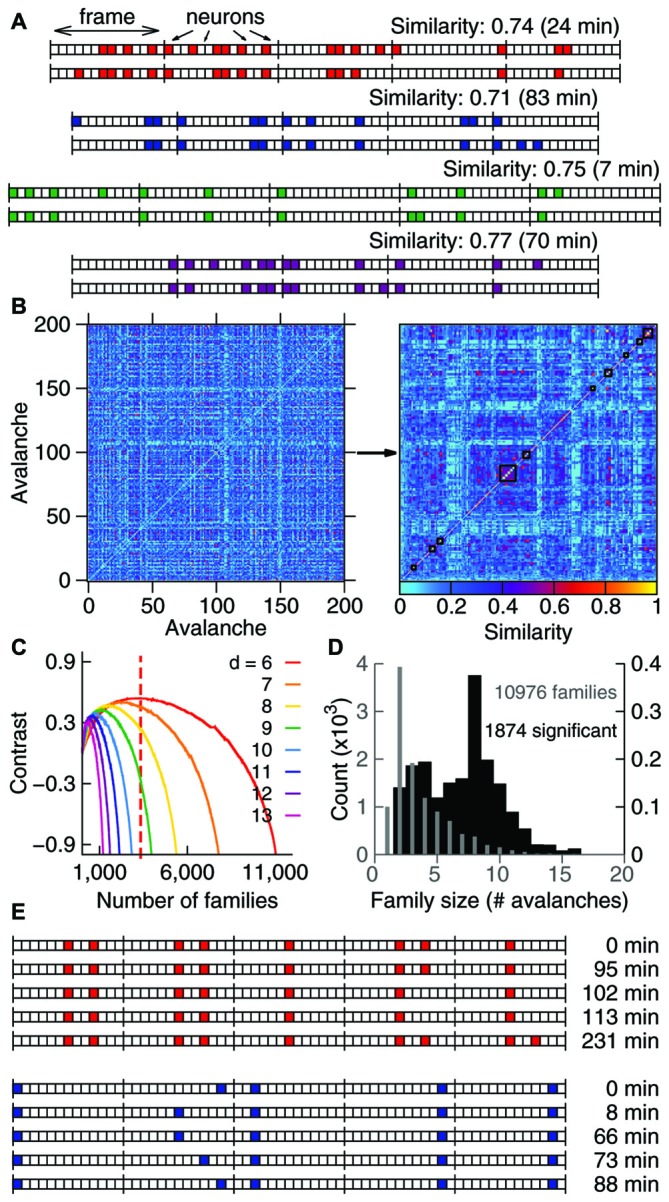
**Avalanches in freely-behaving animals comprise families and recur above chance. (A)** Avalanches represented as binary vectors in which colors indicate spike activity. Depicted are representative examples from four different samples of similar avalanche pairs separated by several minutes (interval of recurrence indicated on the right). **(B)** Similarity matrix for 200 representative avalanches from one sample, before and after clustering (left and right panels, respectively). Black lines indicate 10 families formed by similar avalanches. **(C)** Contrast function for one sample (colors represent different avalanche durations), with the maximum for one duration indicated by the dashed line. **(D)** Number of families of each size for one sample. Gray indicates all families detected (left *Y* axis); black indicates significant families (right *Y* axis). Note that the *Y* axes are scaled by a factor of 1000. **(E)** The recurrence over time of highly similar avalanches defines avalanche families. For the two representative families shown, the occurrence time is relative to the first observed avalanche.

In order to compare avalanches we converted them into one-dimensional vectors. This was done by turning each frame into a vector with dimension equal to the number of recorded neurons and then concatenating those vectors (see Figures 1A,E). Therefore, each avalanche was coded as a sequence of 0s (when said neuron did not fire in said frame) and 1s (when the neuron did fire). This allowed us to compare avalanches of the same duration (number of frames) by a Boolean similarity measure: *Sim(A,B)* = |*A* ∩ *B*| / |*A* ∪ *B*|, whose value ranged from 0 (least similar) to 1 (identical avalanches). In other words, this measures the summed number of spikes from the same neuron and frame divided by the total number of unique spikes in both avalanches (e.g., the first pair of avalanches in Figure 1A had 14 coincident firings and a total of 19 unique activations, leading to a similarity value of 14/19 = 0.74). To avoid temporal misalignment we employed a shift procedure. We compared each pair of avalanches in two additional ways: matching the n-th frame of avalanche A to the (n − 1)-th frame of avalanche B (the first frame of avalanche A is compared to a blank frame) and matching the n-th frame of avalanche A with the (n + 1)-th frame of avalanche B (the last frame of avalanche A is compared to a blank frame). In this case the similarity between A and B was the maximum value when all possible shifts were considered (−1, 0 and +1). Employing this procedure has not led to a significant change in the results found.

With this method we could then compute a similarity matrix by comparing all pairs of avalanches available (note that *Sim*(*A, B*) *= Sim*(*B, A*) and *Sim*(*A, A*) = 1). The produced matrix tells us how similar any two avalanches from the set are (see Figure 1B). Note that we have a similarity matrix for each avalanche duration analyzed, since we only compared pairs of avalanches of the same duration. A minimum duration of three frames was imposed, to avoid comparison between overly simplified patterns.

Due to the computational cost involved in calculating and storing the similarity matrices, we also discarded avalanches of a given duration from the analysis when more than 15,000 of them were observed. Table 1 shows the minimum avalanche duration used for each sample. No qualitative differences between samples with and without discarded avalanches were observed.

### Clustering Algorithm

We implemented a greedy paired clustering algorithm in order to sort the similarity matrices. The method consisted of pairing the most similar avalanches in each step of the algorithm, until all avalanches grouped into a single cluster. The optimum clustering point was obtained through a contrast function, defined as: *C* = (*S*_in_ − *S*_out_)/(*S*_in_ + *S*_out_), where *S*_in_ is the average among clusters of the mean similarity within the clusters (measuring the average similarity inside clusters) and *S*_out_ is the average among pairs of clusters of the mean similarity between the clusters (measuring the average similarity outside clusters). This contrast function was calculated for every step of the algorithm, and its peak gives the most distinct grouping of clusters that is possible to assemble from the data set (see Figure 1C; note that different durations have different contrast functions, leading to different peaks). We refer to these optimum clusters as families of avalanches (see Figure 1E). Note that all avalanches that group into a family must have the same duration, since each duration produces a separate similarity matrix and therefore a separate set of clusters. For the subsequent analysis, families of different durations were pooled together. We did not observe any dependency of the results with avalanche durations.

### Family Significance

To measure the statistical significance of each family, we compared its size (number of members) and average similarity (between its members) with what would be expected by chance. In order to achieve that, we created a shuffled dataset by randomly permuting the order of active frames and then permuting the active neurons in each active frame. This shuffling method was implemented to obtain a chance estimate of both the spatial and temporal structure. Note that this procedure preserves the total number of spikes (and hence the average firing rates) as well as the avalanche durations.

For each of the 100 shuffled datasets considered we applied the same procedure employed in the original data to obtain families of (shuffled) avalanches. We then calculated the probability of obtaining a shuffled family with a given size and average similarity. This probability defined a *p* value for each original family separately. In other words, the *p* value for a family of size N and average similarity S is given by the probability of finding a shuffled family with size N and average similarity not smaller than S.

Since we had to assess the significance of thousands of families, we needed a multiple comparisons correction. We chose to implement the Benjamini-Hochberg procedure (Benjamini and Hochberg, 1995) to avoid an excessive loss of true positive cases in exchange for no false positives. In brief, the method consists of comparing each *p* value to the Benjamini-Hochberg critical value, $\frac{r}{n}Q$, where *r* is the rank of the *p* value (1 meaning the smallest value), *n* is the number of values (or in our case families) and *Q* is the false discovery rate desired. The largest *p* value that is below the Benjamini-Hochberg critical value is considered significant, and so are all the *p* values smaller than this one. We set *Q* = 0.1 which means that the estimate fraction of families considered significant by chance is around 10%. Note that 3 out of 12 samples did not yield any significant families with the false discovery rate employed, and were excluded from the analysis (see Table 1). By further reducing the *Q* value more samples are excluded. In order to achieve a good compromise between a more rigorous statistical test and a larger number of samples we chose *Q* = 0.1. No qualitative changes in the results were observed when employing *Q* values from 5% to 25%. Table 1 shows the number of significant families and the maximum *p* value that is considered significant, together with the estimated number of false positives, for each sample.

### Significance of the Relation Between Avalanche Patterns and Behavior

**Figure 2:**
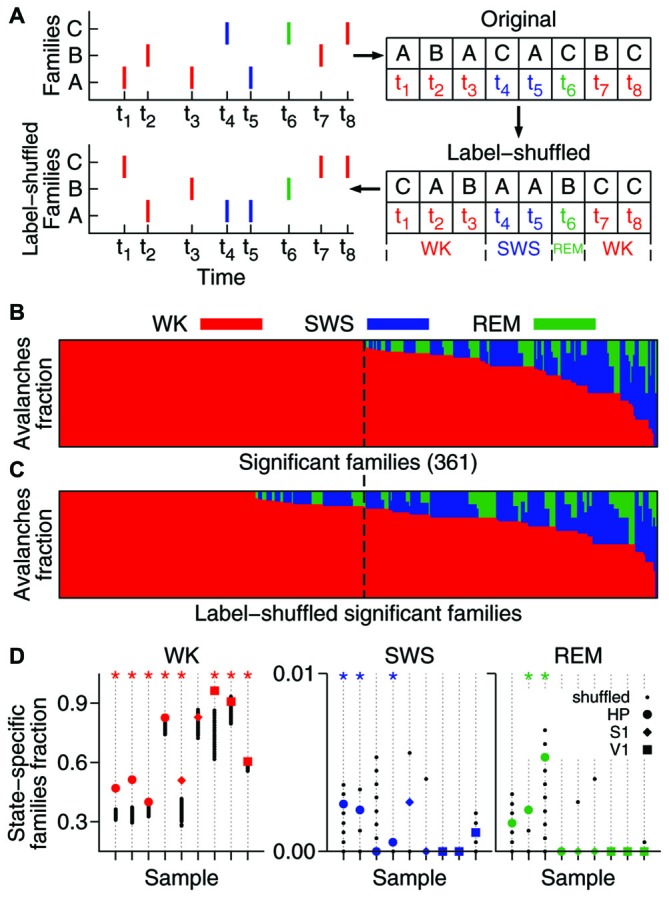
**A significant fraction of the avalanche repertoire occurs exclusively during waking. (A)** To assess the significance of the results, significant families were subjected to the shuffling of family labels. This procedure preserves the distribution of family sizes (see Figure 1D), the number of avalanches in each behavioral state, and the times of occurrence of each avalanche. **(B)** Fraction of avalanches occurring during waking (WK), slow-wave sleep (SWS) and rapid-eye-movement sleep (REM) for each of the 361 significant families of one sample (ranked by WK and then SWS prevalence). The dashed line indicates the boundary that separates waking-specific families from other families. **(C)** Same as in **(B)**, but for families obtained from one single label-shuffled set. Note that many shuffled families comprising multiple states fall to the left of the dashed line, indicating that the number of the non-shuffled families that are waking-specific is larger. **(D)** For each data sample (animal/brain region), the fraction of state-specific families is compared to 1000 shuffled sets. Asterisk indicates significant results (*p* = 0.05). While the state specificity of families that occur during sleep is limited (3/9 samples in SWS and 2/9 in REM), waking-specific families occur significantly in 8/9 samples.

**Figure 3:**
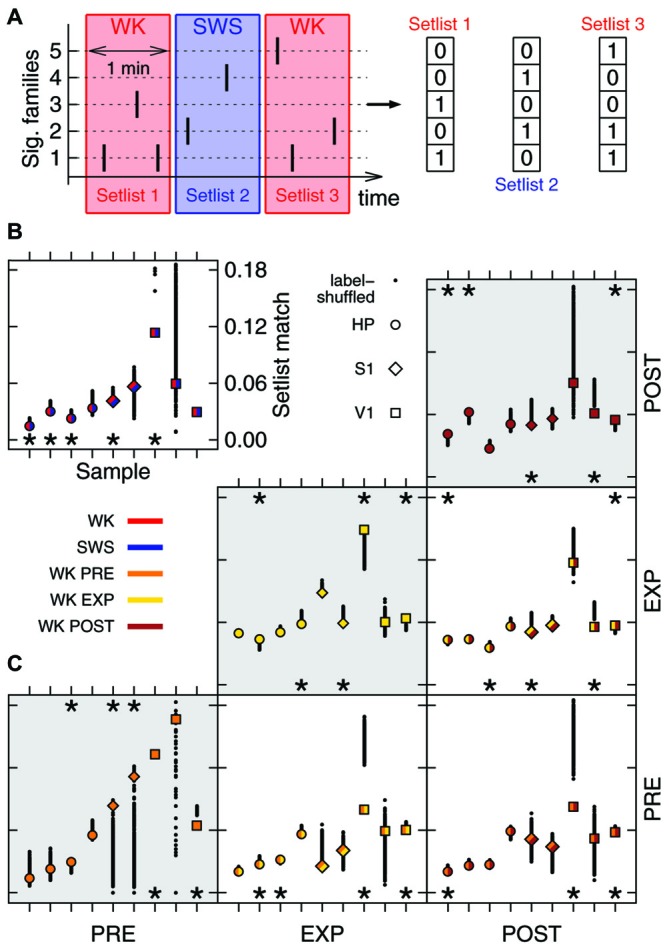
**Family repertoire is behavior-dependent. (A)** Setlists are defined as binary vectors composed of 1s (or 0s) for each significant family observed (or absent) in a given 1 min time window. **(B)** Mean similarity between WK and SWS setlists (label-shuffled data shown in black). Significant cases (*p* = 0.025) are indicated by asterisks on top (similar setlists) or bottom (dissimilar setlists) of the plot. All data samples represented; symbols indicate brain region, colors indicate condition. **(C)** Same as in **(B)**, now comparing WK setlists through different experiment stages (PRE, EXP and POST). Gray areas indicate comparisons within the same stage.

**Figure 4:**
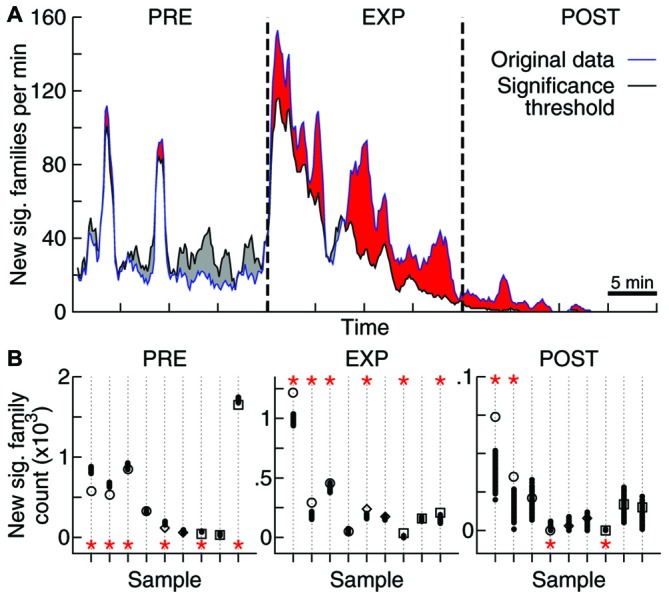
**Objects exploration affects repertoire growth. (A)** Rate of new significant families observed as function of time. Original data for one sample (blue) as well as the threshold for significance obtained through 1000 label-shuffled sets (black) are shown, with PRE, EXP and POST periods indicated. Red areas represent periods of significant generation rate (*p* = 0.05). **(B)** Total number of families that appeared first in each experiment stage, for each data sample (same order as in Figures 2, 3; open symbols: original data, solid circles: label-shuffled sets). Significant cases (*p* = 0.025) are indicated by asterisks on top (high rate) or bottom (low rate) of the plot.

**Figure 5:**
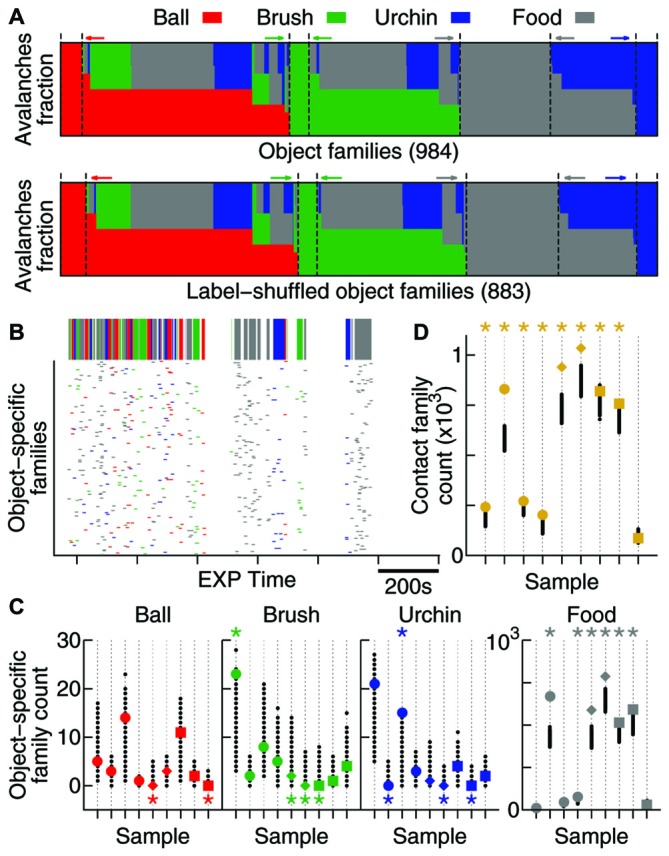
**Contact specificity is observed in avalanche patterns. (A)** Fraction of avalanches during EXP that occurred while the animal was in contact with each object (coded by colors). Note that the original data (top) and the label-shuffled dataset (bottom) profiles are similar, but the total number of families differs. Dashed lines and colored arrows emphasize families that are specific to a particular object. **(B)** Time occurrence of avalanches from families which are specific to one object. In detail above, the color-coded bar shows contact windows for each object. **(C)** Number of object-specific families observed (see “Results” Section). All samples are shown (same order as in previous figures, in colors), together with 1000 label-shuffled sets (in black). Significant cases (*p* = 0.025) are marked by asterisks on top (high number) or bottom (low number) of the plot. **(D)** Same as in **(C)**, but for contact families or, in other words, families exclusively composed of avalanches that occurred during contact with any object.

In order to calculate the statistical significance of the results relating families to behavior, we implemented a different type of shuffling. By reassigning each avalanche to a random family, while preserving family sizes and the behavioral state associated to each avalanche, we create a data set in which families and behavior are decorrelated. We called this procedure label-shuffling, since the only property of the avalanches that changed was the family label. Label-shuffling was used to assess the significance of the results shown in Figures 2, 3, 4, 5. The associated *p* values were obtained by calculating the probability that a label-shuffled set presents the same (or better) characteristics compared to the original data. For example, to assess the significance of the number of waking-specific families in the original set, this number was computed for each label-shuffled set. The fraction of those sets whose number of waking-specific families was equal or higher than the one obtained from the original data is the *p* value. Significance was defined at a *p* = 0.05 level (or *p* = 0.025 for two-sided tests). Note that in this case we do not have a problem of multiple comparisons since each sample is compared only once.

To assess the significance of the results for the entire set of experiments, by considering the *p* values of all samples analyzed, we employed Fisher’s (1950) method, which consists in evaluating the odds of obtaining a set of *p* values by chance in independent tests with the same null hypothesis. If the null hypothesis is true, one should obtain a uniform distribution of *p* values in a series of independent tests. By taking the logarithm of each *p* value, that supposedly uniform distribution is transformed into an exponential distribution. After scaling that value by a factor of 2, a chi-squared distribution with two degrees of freedom is obtained. Adding *k* independent chi-squared values, each with two degrees of freedom, results in a chi-squared distribution with *2k* degrees of freedom, which can then be tested using chi-squared statistics: $\chi_{2k}^{2}=\sum_{i=1}^{k}\ln(p_{i})$, where in our case *k* is the number of samples being tested and *p_i_* is the *p* value of the sample *i*.

### Avalanche Patterns Repertoire

To evaluate how the repertoire of avalanche patterns evolve with time we defined what we called setlists: the subset of patterns that were recruited during 1 min windows, represented by one-dimensional vectors containing 1 for significant families that were present during that window and 0 for those that were not. Setlists were defined separately for each behavioral state (WK and SWS) and stage of the experiment (PRE, EXP and POST). These windows are not necessarily contiguous: one setlist could be composed of the final 30 s of a SWS episode plus the first 30 s of the next SWS episode. The reason for this is that there are not enough contiguous 1 min windows of SWS for good statistics, and if we decrease the window length there are not enough significant families recruited for good statistics. Even in this case, REM was not included because its total amount did not yield enough setlists for a relevant statistical comparison. The number of setlists obtained for each condition is showed in Table 1.

The same measure employed to compare avalanches was used to calculate how similar each pair of setlists are (note that both avalanches and setlists are represented by one-dimensional binary vectors). Label-shuffling was used to assess the significance of the results. In the case of setlists comparison two conditions can be either significantly similar or dissimilar (or not significantly different).

## Results

### Spike Avalanches in Freely-Behaving Rats Constitute Spatiotemporal Activity Patterns

In order to investigate whether spike avalanches are grouped into distinct spatiotemporal patterns we represented them as binary vectors of concatenated time bins of duration Δt (frames) in which every slot indicates the state of one neuron (in Figure 1A colored slots indicate spike occurrence). We defined avalanche similarity as Beggs and Plenz (2004): the number of coincident activations shared by two given spike avalanches (of the same duration) for each bin, normalized by the total number of co-activations. The left panel in Figure 1B shows a similarity matrix for 200 spike avalanches. The right panel in Figure 1B shows the same data after hierarchical grouping by a paired clustering algorithm. Similar avalanches are grouped along the matrix diagonal, indicating that *in vivo* spike avalanches assemble as distinct groups whose members are more similar to each other than to those outside the group.

Inspection reveals that some spike avalanches recorded many minutes apart from each other in fact show great similarity (Figure 1A). To determine the best partitioning of the avalanche set, we calculated a contrast function that measures how distinct the groups are from each other at every step of the algorithm. Groups obtained at the maximal contrast (see Figure 1C) defined families of avalanches that were stable and repeatable throughout the 4–5 h recordings (Figure 1E). To check whether spike avalanche families could occur by chance, we calculated the probability (or *p* value) of obtaining a family with a given size and average similarity by comparing them with families obtained from 100 datasets with shuffled spikes (Stewart and Plenz, 2006). We then implemented the Benjamini-Hochberg procedure (Benjamini and Hochberg, 1995) and set the false discovery rate as 10% in order to determine which families are significant (see “Materials and Methods” Section). In each sample (animal/brain region), thousands of families were detected (13,772 ± 2130), of which hundreds were significant (599 ± 680, Table 1), with an average of ~8 avalanches per significant family (Figure 1D). We therefore conclude that spike avalanches recorded in behaving rats recur over time in a highly stereotyped manner, similarly to what was observed in reduced preparations (Beggs and Plenz, 2004).

### Spike Avalanche Patterns are Related to Behavioral States

We investigated the behavioral specificity of such repeatable patterns by calculating for each family the fraction of avalanches that occurred within WK, SWS and REM (Gervasoni et al., 2004). A substantial proportion of significant families (~40–90%) were exclusively comprised of avalanches that occurred during WK. In contrast, spike avalanches observed during SWS or REM typically belonged to families that were also represented during WK. Given the prevalence of WK across the sleep-wake cycle (Gervasoni et al., 2004), one could argue that it is not surprising that WK harbors more patterns. To control for this imbalance, we shuffled the family labels of the avalanches (Figure 2A).

The label-shuffling procedure preserves family sizes and the behavioral state associated with each avalanche, but destroys the correlation between families and behavior by randomizing the identity of the members of each family. For example, consider family B in Figure 2A (top). Avalanches 2 and 7, which comprise family B, both occurred while the animal was awake (at times t_2_ and t_7_, respectively). That makes family B WK-specific. However, after label-shuffling, avalanche 2 was assigned to family A, and avalanche 7 to family C. Avalanches 3 and 6 were assigned to family B, but since avalanche 6 occurred while the animal was in REM, family B is no longer WK-specific. On the other hand, family C became WK-specific after label-shuffling. Note that the number of avalanches in each family is the same before and after shuffling. Furthermore, since only family labels are being shuffled, the number of avalanches in each behavioral state (the avalanche identity) is also conserved.

Therefore, this method allows asking whether there is a correlation between avalanche patterns and behavior. As can be seen by comparing Figure 2B (original data, one sample) and Figure 2C (label-shuffled data, one set), this procedure led to a smaller number of WK-specific patterns in the shuffled dataset. For each data sample, Figure 2D shows the fraction of significant families that were state-specific, in comparison with 1000 label-shuffled sets. A statistically significant number of waking-specific patterns (*p* = 0.05) was detected in eight out of nine samples, which leads to a significant result for the entire set of experiments (*p* < 10^−5^; see “Materials and Methods” Section). Although SWS-specific patterns were marginally observed (3/9 samples), the results for the entire set of experiments were significant (*p* = 0.01). In contrast, REM-specific patterns did not occur in a significant number (2/9 significant samples). Interestingly, when we sorted the samples by brain region, a different picture arose: for the HP, all states provided a significant number of specific patterns (WK: *p* < 10^−5^; SWS: *p* < 10^−3^; REM: *p* = 0.002), while for the neocortical areas only waking did so (S1: *p* = 0.001; V1: *p* < 10^−5^).

The hundreds of significant families found in an entire recording compose a repertoire of avalanche types. However, only a subset of this repertoire, hereby named setlist, can be observed within a limited time window. Typically, ~20% of the families of a repertoire were observed within the setlist of any 1 min window. Let a 1 min setlist be represented by the binary vector containing 1 for every present family and 0 otherwise (Figure 3A). We calculated the average similarity among all pairwise setlist combinations, classifying each setlist according to behavioral state (WK and SWS) and stage of experiment (PRE, EXP and POST). REM data was not included because its total amount did not yield enough setlists for a relevant statistical comparison (see “Materials and Methods” Section). The results were then compared to those obtained with label-shuffled datasets.

Figure 3 shows the average similarity between setlists of different types, with label-shuffled datasets in black. Note that a setlist similarity value above/below the ones obtained from the shuffled sets indicates that the setlists compared are more/less similar than expected by chance. Significant cases (*p* = 0.025) are indicated by asterisks. In Figure 3B, WK setlists are compared to SWS ones. The significant dissimilarities observed (5/9 samples; *p* < 10^−5^) indicate that although the SWS repertoire of avalanche families typically overlaps with that of WK (Figure 2B) the actual recruitment of patterns within restricted periods of time is much more dissimilar than what chance would predict.

### Contact with Novel Objects Gives Rise to Specific Spike Avalanche Patterns

Comparing WK repertoires from different experiment stages reveals an influence of the exposure to novel objects. There is a reasonable amount of homogeneity in the pattern repertoire within PRE (3/9 significantly similar samples, bottom-left box in Figure 3C; *p* = 0.002) and POST (3/9 significantly similar samples, top-right box in Figure 3C; *p* < 10^−3^). However, PRE and POST setlists tend to be dissimilar (3/9 significantly dissimilar samples, bottom-right box in Figure 3C; *p* < 10^−3^), which suggests that EXP changes the regime of avalanche repertoires. Moreover, PRE/EXP and EXP/POST setlist matches are less similar than expected by chance (4/9 and 3/9 significantly dissimilar samples, respectively; *p* < 10^−5^, both cases), raising the question: is there an exclusive set of patterns being recruited during objects exploration?

To tackle this issue, we assessed the effect of novel experience on the rate of emergence of new avalanche patterns. The avalanche repertoire in a given data sample grows at a highly variable rate (Figure 4A). When the objects were presented for free exploration by the animal, the rate of emergence of new patterns was affected: significantly more patterns were generated during EXP than was expected by chance. If avalanche patterns were uncorrelated to novel objects exploration, a lower pattern generation rate during EXP should have been observed (Figure 4A) for most data samples (6/9 samples with significantly higher rate during EXP, Figure 4B; *p* < 10^−5^). Furthermore, a higher rate was expected during PRE (6/9 samples with lower rate; *p* < 10^−5^), and although the results seem confusing for POST (2/9 samples with significantly higher and lower rate) it becomes once again clear after the brain regions are separated for the analyses of significance: the pattern generation rate in HP was significantly higher than expected by chance (*p* < 10^−3^), while in V1 it was significantly lower than expected by chance (*p* = 0.02). The results for S1 were not significant either way.

In order to investigate whether the new families that emerge during EXP specifically relate to individual objects, a mix of objects, or to no objects at all, we defined two types of avalanche families: (1) contact families are those whose members occurred while the animal was in contact with any object (Figure 5A); and (2) object-specific families are the ones whose members occurred while the animal was in contact with a given object (families represented by one color only in Figure 5A). Given the short windows of contact with the objects (Figure 5B, top) and the fact that they were novel to the animals, many of these object-related families may not have had enough time to consolidate into patterns. For that reason, we considered all families observed (including non-significant ones) for this analysis.

The times of occurrence of avalanches from object-specific families are shown in Figure 5B. Note that although the animal spends considerable EXP time exploring the four objects, very few object-specific families are observed with the exception of food, to which the animals typically dedicated more attention. In order to discover whether these few object-specific families can be expected by chance, once again we used 1000 label-shuffled datasets. Figure 5C shows that the number of ball-, brush- and urchin-specific families observed were not significantly high (2 significant cases in 27 possible). In fact, both neocortical areas recorded presented fewer object-specific families than expected by chance (S1: *p*_ball_ = 0.007, *p*_brush_ < 10^−3^, *p*_urchin_ < 10^−3^; V1: *p*_ball_ = 0.02, *p*_brush_ = 0.004, *p*_urchin_ = 0.02). The HP, however, displayed a significantly high number of urchin-specific families (*p* = 0.008), and although the number for the other two objects was not significant, they followed the same trend (*p*_ball_ = 0.11 and *p*_brush_ = 0.15). A significant number of food-specific families was observed for all areas (6/9 significant samples, *p* < 10^−5^).

Moreover, contact families, which indicate avalanche families being shared by more than one object, are present in a significant number for all areas (8/9 samples, Figure 5D; *p* < 10^−5^), suggesting that the new patterns arising during EXP are specifically related to contact with novel objects.

## Discussion

Previous work has shown that LFP avalanches in cortical slice cultures form patterns that are stable for many hours (Beggs and Plenz, 2004). These results, together with the statistical signatures linking the absence of characteristic sizes and durations with a putatively critical brain (Beggs and Plenz, 2003), gave rise to the promising conjecture that neuronal avalanches could play an important role in information processing. Although a number of theoretical works ensued (Bertschinger and Natschläger, 2004; Haldeman and Beggs, 2005; Kinouchi and Copelli, 2006; Rämö et al., 2007; Nykter et al., 2008), no further experimental evidence has been provided to support that hypothesis in non-reduced preparations. To fill this gap, we recorded spiking activity from the HP, as well as primary somatosensory and visual cortices of freely-behaving rats exposed to novel objects, to assess to what extent spike avalanches are influenced by behavior.

Using the same tools that were previously employed to assess recursivity in spatiotemporal patterns of LFP activity *in vitro* (Beggs and Plenz, 2004; Stewart and Plenz, 2006), we confirmed that spike avalanches in freely-behaving rats indeed recur over time, and are grouped in statistically significant families. It is interesting to note that we found important differences between the brain regions recorded in this study, although the number of samples for each area was limited. We found on average 1105 significant families in HP, 152 in S1 (with 2 out of 4 samples contributing no significant families) and 541 in V1 (with 2 out of 4 samples contributing no significant families). More importantly, specific patterns were obtained in a significant number more often for HP than in other areas. This would be in agreement with the hypothesis that these patterns have a role in memory formation, since HP is more strongly associated with it than the other areas.

While we acknowledge the substantial differences between the *in vitro* LFP preparation and our experimental preparation, it is nonetheless interesting to compare some basic numbers. With about four times fewer avalanches analyzed (~10,000) Beggs and Plenz (2004) found on average 30 ± 14 significant families, whereas we have found a number 20 times higher (599 ± 680). It remains to be investigated to which extent the larger number of families we observed results from a richer dynamical structure of the intact brain (in contrast with a slice culture), and to which extent it is a result of intrinsic differences between LFP and spike recordings. It is also interesting to note that the average size of significant families is about three times smaller in our case (~23 vs. ~8), which means that in the *in vitro* preparation the patterns repeat more. It is not clear whether this difference could be explained by spatial (number of neurons, distance between neurons) and/or temporal (total time of recording) sampling limitations, or once again is a consequence of the intrinsic differences between the experimental preparations. It is important to note that none of our results depends on the number of avalanches within a family, and therefore the small size of the families did not have any implications for the analysis.

Previous analysis of this data has shown that the size of spike avalanches in freely-behaving rats has remarkably similar statistical properties across brain regions (HP, S1 and V1), behavioral states (WK, SWS and REM) and stages of the experiment (PRE, EXP and POST). This universal statistical behavior holds not only for size distributions, but also for the temporal structure of avalanche recurrence, whose interval distributions for all possible cases could be described by a single scaling function (Ribeiro et al., 2010). Although the results can be slightly different for other preparations, with small deviations among vigilance states using LFP (Priesemann et al., 2013) or EEG (Meisel et al., 2013), the same kind of universality was observed in other studies in the context of spike avalanches (Friedman et al., 2012; Priesemann et al., 2014).

Despite following the same dynamical rules, what we have shown here is that the identities of the avalanches giving rise to that universality are anything but universal, with important differences among behavioral states. For instance, a significant number of avalanche patterns are specific to WK, whereas avalanches occurring during sleep states typically also occur during WK, although sleep-specific patterns did occur in a significant number in the HP. The lower number of significant cases observed within the sleep states may be explained by the lower amount of time spent in these states, or perhaps this is indeed an intrinsic difference between waking and sleep for the neocortical areas. In any case, the large amount of sleep-state avalanches sharing families with waking avalanches can be interpreted as evidence of reverberation, during sleep, of a subset of the avalanches that occur during WK (Pavlides and Winson, 1989; Wilson and McNaughton, 1994; Ribeiro et al., 2004). This also suggests that, while the rules that govern recruitment of avalanches during sleep preserve most of their statistical properties observed during waking, there should be also some reorganization of these rules to account for the differences in the composition of the avalanche repertoire recruited. The stark differences in neuromodulatory milieu across the sleep-wake cycle (Gottesmann, 2002; Luppi and Fort, 2011) most likely account for such reorganization.

The repertoire of avalanche patterns is homogeneous before novel experience, but changes significantly once novel objects are presented to the rats. Furthermore, the rate at which new patterns are generated during object exposure is significantly higher than what would be expected if avalanches did not contribute to information processing. Moreover, we found evidence of object encoding by shared avalanche patterns.

It is important to assess to which extent the results obtained can be explained by changes in firing rates. Not only those are higher during WK than in the sleep states, but also they are significantly increased when the novel objects are introduced. This non-stationarity could, in principle, lead to different activity patterns, possibly explaining the significant differences found between WK and SWS families or the high pattern generation rate during EXP. A generalized increase in firing rate can affect avalanches in three ways: (1) making them larger; (2) making them longer; (3) making them occur more often. The first two are related to size and duration distributions, respectively. The third is related to the waiting time distribution. The method employed to determine the significance of our results, i.e., the label-shuffling procedure, conserves the duration of the avalanches, as well as their rate of occurrence, but shuffles sizes in a non-trivial way. However, the cross-correlation between the firing rate and the family generation time series is quite weak (0.17 ± 0.12, averaged across all samples). Moreover, the original and shuffled pattern generation time series are strongly correlated (0.89 ± 0.15). Taken together, those results suggest that only a small fraction of the results can be explained by changes in firing rate, and most of those changes are already captured by our shuffling procedure.

The lack of object specificity in S1 and V1, with exception of food (to which the animals dedicated considerably more time), is perhaps not surprising. Instead of encoding specific objects, the avalanche patterns in those areas could be representing features of the objects. This hypothesis is corroborated by the fact that most samples showed a significant number of patterns shared between the different objects. This is also compatible with food being the exception since it is the only object associated with a reward. If such is indeed the case, one could expect that a new object introduced to the animal could be represented by a set of existing patterns related to features that familiar objects share with the new one. Another possible explanation for this lack of specificity may be the very fine temporal scale (up to hundreds of milliseconds) of the avalanches. Vasconcelos et al. (2011) showed that decoding of specific objects is achieved with the same dataset for a much different time scale, one order of magnitude higher. Most likely, avalanches comprise mixtures of underlying assemblies of synchronized neurons (Hebb, 1949) at an even finer time scale (Plenz and Thiagarajan, 2007). Our results are compatible with the notion that the representation of specific objects is not likely to be found at the temporal scale of avalanches, but rather at the scale of their sequences.

In the HP, on the other hand, there is strong evidence in the opposite direction: we found patterns that occur specifically during the contact with one object. It remains to be investigated whether those patterns would resurface in subsequent contacts with now familiar objects. The results so far suggest that those avalanches could represent a substrate of the memory of the objects.

## Funding

This work was supported by Coordenação de Aperfeiçoamento de Pessoal de Nível Superior (CAPES), Financiadora de Estudos e Projetos (FINEP) Grant 01.06.1092.00, Pró-Reitoria de Pós-Graduação da Universidade Federal do Rio Grande do Norte (UFRN), Conselho Nacional de Desenvolvimento Científico e Tecnológico (CNPq)/Ministério da Ciência, Tecnologia e Inovação (MCTI), CNPq Grants 481351/2011-6, 306604/2012-4, 480053/2013-8 and 310712/2014-9, Programa de Apoio a Núcleos Emergentes (PRONEM) 003/2011 FAPERN/CNPq and PRONEM 12/2010 FACEPE/CNPq, Pew Latin American Fellows Program in the Biomedical Sciences, Capes SticAmSud, FAPESP Center for Neuromathematics (Grant #2013/076990, São Paulo Research Foundation), and NIMBIOS working group “Multi-scale analysis of cortical networks”.

## Author Contributions

TLR performed data analysis; SR performed recordings; MC, SR and TLR designed the study; MC, SR and TLR wrote the article.

## Conflict of Interest Statement

The authors declare that the research was conducted in the absence of any commercial or financial relationships that could be construed as a potential conflict of interest.
